# Telomeric repeat diversity across Nepomorpha (Hemiptera, Heteroptera) revealed by whole-genome sequencing data

**DOI:** 10.3897/compcytogen.20.198500

**Published:** 2026-07-22

**Authors:** Desislava Stoianova, Gayane Karagyan, Snejana Grozeva

**Affiliations:** 1 Institute of Biodiversity and Ecosystem Research, Bulgarian Academy of Sciences, 1 Tsar Osvoboditel Blvd, 1000 Sofia, Bulgaria Scientific Center Zoology and Hydroecology – NAS RA Yerevan Armenia; 2 Scientific Center Zoology and Hydroecology – NAS RA, 7, P. Sevak str., 0014 Yerevan, Armenia Institute of Biodiversity and Ecosystem Research, Bulgarian Academy of Sciences Sofia Bulgaria

**Keywords:** Chromosome evolution, k-mer-based telomere repeat identification, phylogenetic signal, (TTAGG)n telomere

## Abstract

Telomeric sequences in the insect infraorder Nepomorpha (Hemiptera: Heteroptera) have previously been characterized only in the basal superfamily Nepoidea, where the canonical arthropod motif (TTAGG)n was confirmed by fluorescence *in situ* hybridization (FISH). The telomeric repeat composition of the remaining Nepomorpha superfamilies remained unknown. Using TREW, a tool for *de novo* identification of candidate telomeric sequences from short-read whole-genome sequencing data, we screened 32 publicly available NCBI datasets representing 24 genera and all 13 Nepomorpha families. The method was validated against species with known (TTAGG)n telomeres in Nepoidea, successfully recovering the expected motif. In Corixoidea, (TTAGG)n was uniformly identified as the best candidate for telomeric sequence across all eight species of three families – Corixidae, Diaprepocoridae and Micronectidae. In contrast, derived 10-bp motifs were recovered from the remaining four superfamilies: (TGGTTAGTGA)n and (TGGTTAGTGT)n in Ochteroidea, (TGGATAGGTG)n and (TGGATAGGAG)n in Naucoroidea (including Aphelocheiridae and Potamocoridae), and (TGGATAGGGT)n uniformly across Notonectoidea and Pleoidea. All derived decamers except those from Ochteroidea share the core octamer TGGATAGG. The retention of (TTAGG)n in the two basal superfamilies and the occurrence of derived decamers in more derived clades are congruent with most current phylogenetic hypotheses for Nepomorpha and support independent evolution of 10-bp motifs within the infraorder. The presence of an identical candidate for telomeric sequence – (TGGATAGGGT)n in Notonectoidea and Pleoidea provides additional support for their close phylogenetic relationship and might be a synapomorphy for this clade. These findings extend the known pattern of telomeric diversification within Heteroptera to Nepomorpha and demonstrate the utility of short-read archival data for taxonomic surveys of telomeric composition.

## Introduction

In most of the eukaryote organisms telomeres cap chromosome ends and protect genomic integrity through tandem DNA repeats maintained by telomerase. It is a quite common mechanism of telomere maintenance, but it is not universal. The pentanucleotide (TTAGG)n is the predominant telomeric motif across arthropods, including most insects (Sahara et al. 1999; Frydrychová et al. 2004; Vítková et al. 2005). However, this ancestral motif has been repeatedly lost during insect diversification, and several lineages now harbour derived telomeric sequences whose identity often remains unknown (Kuznetsova et al. 2020; Lukhtanov and Pazhenkova 2023).

The order Hemiptera comprises more than 100,000 species, including true bugs (Heteroptera), cicadas and relatives (Auchenorrhyncha), aphids, whiteflies, psyllids and scale insects (Sternorrhyncha), and moss bugs (Coleorrhyncha); in the suborders Sternorrhyncha, Auchenorrhyncha and Coleorrhyncha only the canonical (TTAGG)n motif has so far been reported (Kuznetsova et al. 2020; Stoianova et al. 2025). In contrast, the suborder Heteroptera exhibits remarkable heterogeneity: while basal infraorders retain the ancestral motif, derived lineages within Pentatomomorpha, Cimicomorpha and Gerromorpha possess non-canonical 10-bp repeats such as (TTAGGGATGG)n, (TTAGGGGTGG)n, (TTAGGGTGGT)n and (TTAGAGGTGG)n (Lukhtanov and Pazhenkova 2023; Kuznetsova et al. 2025; Stoianova et al. 2024, 2025). The occurrence of derived motifs across multiple infraorders suggests that telomeric sequences have undergone repeated modification within Heteroptera.

The infraorder Nepomorpha (Heteroptera), commonly known as true water bugs, is a diverse group, comprising nearly 2,400 described species (Schuh and Weirauch 2020), although family- and superfamily-level classifications vary among authors. Hebsgaard et al. (2004) recognized 11 families distributed across seven superfamilies, treating Corixidae as a single family and proposing Aphelocheiroidea (Aphelocheiridae + Potamocoridae) as a new superfamily distinct from Naucoroidea. Subsequent studies have split Corixoidea into three families – Corixidae, Diaprepocoridae and Micronectidae – resulting in 13 families (Ye et al. 2020; Wang et al. 2021). The number of superfamilies also differs: Ye et al. (2020) uses classification with these 13 families divided into six superfamilies, with Naucoroidea including Aphelocheiridae and Potamocoridae. Later Wang et al. (2021) recognized only five by treating Pleidae and Helotrephidae as a part of Notonectoidea rather than as a separate superfamily Pleoidea. The same divisions were suggested much earlier by Schuh and Slater (1995). In the present study, we follow the classification of Ye et al. (2020), with six superfamilies (Nepoidea, Corixoidea, Ochteroidea, Naucoroidea, Notonectoidea and Pleoidea) and 13 families, as this scheme facilitates comprehensible presentation of telomeric variation across taxonomic levels.

To date, telomeric sequences have been characterized only within the basal superfamily Nepoidea. Fluorescence *in situ* hybridization (FISH) confirmed the presence of (TTAGG)n in six *Belostoma* Latreille, 1807 species: *B. candidulum* Montandon, 1903, *B. dentatum* Mayr, 1863, *B. elegans* Mayr, 1871, *B. elongatum* Montandon, 1908, *B. micantulum* Stål, 1860 and *B. oxyurum* Dufour, 1863 (Chirino et al. 2017), as well as in *Lethocerus patruelis* (Stål, 1854) (Kuznetsova et al. 2012). Within the family Nepidae, the canonical motif was similarly detected in *Nepa cinerea* Linnaeus, 1758 and *Ranatra linearis* (Linnaeus, 1758) (Angus et al. 2017). No telomeric sequence data are currently available for the remaining five Nepomorpha superfamilies.

Historically, telomeric sequences in insects have been identified through molecular cytogenetic approaches. Southern blot hybridization using labelled oligonucleotide probes provided the first evidence of telomeric repeat composition in many species (Sahara et al. 1999; Frydrychová et al. 2004). FISH with telomeric probes subsequently became the standard method for mapping telomeric sequences directly onto chromosomes, enabling both testing of potential telomeric motifs and localization of interstitial telomeric sites (Kuznetsova et al. 2020, 2025). However, these experimental approaches require known or inferred candidate sequences.

The advent of next-generation sequencing (NGS) has enabled computational approaches for *de novo* telomere identification. Early bioinformatic tools, such as Sequence Repeat Finder (SERF), identified candidate telomeric repeats from NGS reads by detecting overrepresented tandem sequences (Peška et al. 2015). More recently, tools designed for chromosome-level genome assemblies have emerged, tidk (Brown et al. 2025) for telomere-specific analysis of assembled genomes, and quarTeT (Lin et al. 2023) for comprehensive telomere-to-telomere genome characterization. Stoianova et al. (2025) applied such genome assembly-based approaches to characterize telomeric sequences across multiple hemipteran suborders. Recently, Telomeric Repeat motif Estimation tool with Whole-genome sequencing data (TREW) (Ryu 2024; Ryu and Kim 2024) was developed specifically for *de novo* identification of telomeric repeat motifs from short-read whole-genome sequencing data, using k-mer counting and within-read consistency checks to identify candidate motifs without requiring prior sequence knowledge.

Here, we employed TREW to screen publicly available short-read whole-genome sequencing datasets from NCBI for Nepomorpha species of 24 genera, representing all 13 families and the six superfamilies. First, we validated the approach using datasets from species with previously characterized (TTAGG)n telomeres: *Nepa cinerea* and *Ranatra linearis* (Angus et al. 2017), and *Lethocerus indicus* (Lepeletier et Serville, 1825), from the genus of the already studied *L. patruelis* (Kuznetsova et al. 2012). Following the validation, we applied the method to identify candidate telomeric motifs in species from the five previously unstudied superfamilies. Our objectives were to assess whether the canonical (TTAGG)n motif is uniformly present across Nepomorpha or whether derived motifs have evolved in particular lineages, thereby contributing to the understanding of telomeric sequence evolution within Heteroptera. The chromosomes of Nepomorpha, like those of all Heteroptera, are holokinetic (Ueshima 1979), meaning that they lack localized centromeres, and the only criterion for identifying individual chromosomes in the karyotype is their size. Therefore, telomeric motifs could be useful as chromosomal markers in comparative analysis of karyotypes.

## Material and methods

### NCBI datasets

We downloaded all available Nepomorpha whole-genome short-read FASTQ datasets from the NCBI Sequence Read Archive (SRA; accessed 26 January 2026). Only datasets with AvgSpotLen ≥ 300 bp were retained. This filtering was applied to improve the reliability of candidate telomeric array detection. After filtering, 32 short-read FASTQ datasets remained (run IDs and metadata are provided in Suppl. material 1: table SS1), representing 24 genera and 13 families.

Among the retained datasets, five represented lineages for which TTAGG telomeres have been reported in the literature. Specifically, TTAGG has been reported for *Nepa cinerea* and *Ranatra linearis* (Angus et al. 2017), and our dataset included three datasets for *N. cinerea* and one dataset for *R. linearis*. In addition, we included one dataset for *Lethocerus indicus*. Although TTAGG has not been reported for *L. indicus*, it has been reported for another species of the same genus (Kuznetsova et al. 2012). We used these five datasets as controls to assess whether TREW can recover the expected telomeric motif in Nepomorpha short-read data. For the remaining species, no published telomeric-sequence data are available, either for the focal species or for other species within their respective families.

### Identification of the best candidates for telomeric repeat motifs

Each dataset was analysed with TREW 0.5.0 (Ryu 2024; Ryu and Kim 2024), a tool developed specifically for *de novo* identification of telomeric repeat motifs from short-read whole-genome sequencing data without requiring genome assembly. This makes it suitable for screening archival datasets deposited in public repositories, where raw sequencing reads are typically available, but chromosome-level assemblies are not. For most Nepomorpha species in this study, no assembled genomes exist; only raw short-read data are available in the NCBI Sequence Read Archive. Single-end datasets were analysed with TREW using, the following: trew short 5 10 <FASTQ.gz> -t 16, whereas paired-end datasets were analysed using TREW’s paired-end mode: trew short 5 10 --paired_end --fq1 <R1.fastq.gz> --fq2 <R2.fastq.gz> -t 16. The TREW software reads each FASTQ record, extracts the nucleotide sequence from every read, and scans it with sliding k-mer windows for all k between the specified minimum and maximum, counting canonicalized k-mers (accounting for cyclic rotations and reverse complements) within reads. For every k, it identifies the most frequent non-low-complexity k-mer and evaluates its frequency against a baseline threshold; only motifs meeting the high-stringency criteria are reported in the “H:” output. For each reported motif, TREW provides: (i) the number of repeat occurrences on the predominant strand (“number of repeat”), (ii) the number of occurrences on the reverse-complement orientation (“number of reverse repeat”), and (iii) the number of occurrences supported in both orientations/ends under the internal consistency checks (“number of pure repeat”). For motif selection per dataset, we inspected the “H:” results, filtered the results, retaining only motifs with five or ten nucleotides – only a pentamer and various decamers have been reported for Heteroptera so far (Stoianova et al. 2024, 2025; Kuznetsova et al. 2025). This filtering was also applied to avoid interpreting shorter k-mers corresponding to internal windows of a given longer tandem repeat as independent telomeric motifs. However, we inspected the candidate motifs of other lengths within the analysed range but none of them showed a more consistent or biologically interpretable pattern than the 5-bp and 10-bp motifs. As a next step we extracted the top three motifs reported by TREW (among the 5-bp and 10-bp motifs) and then chose as the final candidate telomeric motif the one with the highest “number of pure repeat”, treating this as the most conservative indicator of a robust, strand-consistent tandem repeat signal within reads.

Because for tandem repeat motifs different reading starting positions yield equivalent sequences (e.g., TGGATAGGGT and TTGGATAGGG are cyclic permutations of the same repeat unit), we report the permutation that best illustrates sequence similarities among the motifs recovered from the datasets in the study.

## Results

TREW analysis of 32 FASTQ datasets, representing 24 Nepomorpha genera, yielded candidate telomeric motifs for all six superfamilies (Table 1, Suppl. material 1: table SS1). In Nepoidea, TREW recovered the canonical (TTAGG)n motif from all six datasets: *Appasus japonicus* Vuillefroy, 1864 and *Lethocerus indicus* (Belostomatidae), three datasets of *Nepa cinerea* and one of *Ranatra linearis* (Nepidae). The results for three of the species served as positive controls, as (TTAGG)n was previously reported for *N. cinerea*, *R. linearis* and a congener of *L. indicus*. Here we report (TTAGG)n in *Appasus* Amyot et Serville, 1843 for the first time.

**Table 1.** Candidate telomeric motifs identified using TREW. NPR (Number of Pure Repeat) – number of repeat occurrences supported in both orientations/ends under TREW’s internal consistency checks; NPRR (Number of Pure Repeat Ranking) – rank position of the reported motif among the top three candidates extracted from TREW’s high-stringency (“H:”) output for that dataset, ranked by number of pure repeat.

**Taxon**	**NCBI SRA accession**	**Top candidate sequence**	**NPR**	**NPRR**
**Superfamily Nepoidea**				
Family Nepidae				
*Nepa cinerea* Linnaeus, 1758	SRR11470103	TTAGG	1057121	top1
*Nepa cinerea* Linnaeus, 1758	SRR13212381	TTAGG	102998	top1
*Nepa cinerea* Linnaeus, 1758	SRR34575523	TTAGG	102998	top1
*Ranatra linearis* (Linnaeus, 1758)	SRR34564609	TTAGG	50663	top1
Family Belostomatidae				
*Appasus japonicus* Vuillefroy, 1864	SRR15404221	TTAGG	0*	top1
*Lethocerus indicus* (Lepeletier et Serville, 1825)	SRR34512194	TTAGG	1685549	top1
**Superfamily Corixoidea**				
Family Corixidae				
*Callicorixa gebleri* (Fieber, 1848)	SRR34512195	TTAGG	655134	top1
*Cymatia bonsdorffii* (Fieber, 1864)	SRR34564612	TTAGG	2523	top1
*Hesperocorixa castanea* (Thomson, 1869)	SRR34564611	TTAGG	837	top1
*Sigara lateralis* (Leach, 1817)	SRR24800582	TTAGG	3039	top1
Family Diaprepocoridae				
*Diaprepocoris barycephalus* Kirkaldy, 1897	SRR34575526	TTAGG	83998	top1
*Diaprepocoris zealandiae* Hale, 1924	SRR13213151	TTAGG	3882148	top1
*Diaprepocoris zealandiae* Hale, 1924	SRR34564610	TTAGG	3882148	top1
Family Micronectidae				
*Micronecta* sp.	SRR34575525	TTAGG	4470	top1
*Tenagobia incerta* Lundblad, 1929	SRR13212379	TTAGG	7002	top1
*Tenagobia incerta* Lundblad, 1929	SRR34575524	TTAGG	7002	top1
**Superfamily Naucoroidea**				
Family Naucoridae				
*Cryphocricos* sp.	SRR34517044	TGGATAGGTG	12639	top1
*Heleolaccocoris ovatus* (Montandon, 1897)	SRR34517046	GGATT	35035	top1
*Limnocoris* sp.	SRR34517043	TGGATAGGAG	58750	top1
*Naucoris scutellaris* Stål, 1860	SRR34517045	TGGATAGGAG	52776	top1
Family Aphelocheiridae				
*Aphelocheirus aestivalis* (Fabricius, 1794)	SRR11470048	TGTGCCTCTC	61220	top1
*Aphelocheirus aestivalis* (Fabricius, 1794)	SRR11470048	TGGATAGGTG	6452	top3
*Aphelocheirus hainanensis* Zettel, 1998	SRR34517042	TGGATAGGTG	16236	top1
Family Potamocoridae				
*Potamocoris hungerfordi* (De Carlo, 1968)	SRR34575522	TGGATAGGTG	6159	top1
*Potamocoris* sp.	SRR13212375	TGGATAGGTG	6159	top1
*Potamocoris* sp.	SRR34517047	TGGATAGGAG	27545	top1
**Superfamily Notonectoidea**				
Family Notonectidae				
*Anisops kuroiwae* Matsumura, 1915	SRR34517041	TGGATAGGGT	30330	top1
*Enithares chinensis* Brooks, 1948	SRR34575521	TGGATAGGGT	357169	top1
**Superfamily Pleoidea**				
Family Helotrephidae				
*Helotrephes semiglobosus* Stål, 1860	SRR34575520	TGGATAGGGT	188127	top1
Family Pleidae				
*Paraplea indistinguenda* (Matsumura, 1905)	SRR34517040	TGGATAGGGT	85257	top1
*Plea minutissima* Leach, 1818	SRR34564608	TGGATAGGGT	34317	top1
**Superfamily Ochteroidea**				
Family Ochteridae				
*Ochterus breviculus* Latreille, 1807	SRR34517048	TGGTTAGTGT	129622	top1
Family Gelastocoridae				
*Gelastocoris* sp.	SRR34517049	TGGTTAGTGA	121480	top1

* For (TTAGG)n in the *A. japonicus* dataset, the number of pure repeats was zero, however, it was the top candidate with substantial support – 21412 repeat counts and 12670 reverse-repeat counts.

In Corixoidea, all ten datasets corresponding to eight species and three families showed (TTAGG)n as the best candidate. Within Corixidae, this included *Callicorixa gebleri* (Fieber, 1848), *Cymatia bonsdorffii* (Fieber, 1864), *Hesperocorixa castanea* (Thomson, 1869) and *Sigara lateralis* (Leach, 1817). The same motif was indicated for Diaprepocoridae: *Diaprepocoris barycephalus* Kirkaldy, 1897 and *D. zealandiae* Hale, 1924 (two datasets); and Micronectidae: *Micronecta* sp. and *Tenagobia incerta* Lundblad, 1929 (two datasets). Here we report (TTAGG)n in Corixoidea for the first time.

Notonectoidea and Pleoidea shared the same derived decamer. Both Notonectidae species (*Anisops kuroiwae* Matsumura, 1915, *Enithares chinensis* Brooks, 1948) returned (TGGATAGGGT)n. The identical motif was recovered from *Helotrephes semiglobosus* Stål, 1860 (Helotrephidae), *Paraplea indistinguenda* (Matsumura, 1905) and *Plea minutissima* Leach, 1818 (Pleidae). Here we report (TGGATAGGGT)n in Notonectoidea and Pleoidea for the first time.

In Ochteroidea, the two sampled families returned very similar decamers: *Gelastocoris* sp. (Gelastocoridae) – (TGGTTAGTGA)n, while *Ochterus breviculus* Latreille, 1807 (Ochteridae) – (TGGTTAGTGT)n. These sequences share the same 10-bp periodicity and differ only by a single nucleotide.

Within Naucoridae, there was variation among species. *Naucoris scutellaris* Stål, 1860 and *Limnocoris* sp. returned (TGGATAGGAG)n, *Cryphocricos* sp. returned (TGGATAGGTG)n, and *Heleolaccocoris ovatus* (Montandon, 1897) returned (GGATT)n. Within Aphelocheiridae, *Aphelocheirus hainanensis* (Zettel, 1998) returned (TGGATAGGTG)n, and in the results for *A. aestivalis* (Fabricius, 1794), a low-complexity decamer (TGTGCCTCTC)n was at the top position, but at third position was (TGGATAGGTG)n. The latter motif was also recovered from two Potamocoridae datasets: *Potamocoris hungerfordi* (De Carlo, 1968) and a *Potamocoris* sp. dataset. The third *Potamocoris* sp. dataset returned the single-nucleotide variant (TGGATAGGAG)n.

## Discussion

Before interpreting the phylogenetic implications of our telomeric data, we must consider which results are the most reliable. The recovery of (TTAGG)n from Nepoidea served primarily as validation of the TREW approach: this superfamily was the only one with prior telomeric molecular-cytogenetic data, and the consistent identification of the expected motif across both families and multiple independent datasets confirms that the method reliably detects telomeric sequences in Nepomorpha short-read data. The novel and most robust findings concern Corixoidea and the Notonectoidea–Pleoidea clade. In Corixoidea, (TTAGG)n was uniformly recovered across all ten datasets spanning eight species and three families – a noticeable consistency that strongly suggests that this represents the true telomeric motif throughout the superfamily. Similarly, the recovery of the derived decamer (TGGATAGGGT)n across all five Notonectidae-Pleidae-Helotrephidae datasets is compelling: such uniformity across phylogenetically distinct lineages is unlikely to result from algorithmic artefacts. Likewise, the decamer (TGGATAGGTG)n was observed in species within each of the three families Naucoridae, Aphelocheiridae and Potamocoridae.

The results for decamers found in single datasets should be interpreted more cautiously. TREW, like all k-mer-based methods, may occasionally report satellite sequences or other repetitive elements rather than true telomeric motifs. The low-complexity decamer (TGTGCCTCTC)n and the pentanucleotide (GGATT)n recovered as top results for *Aphelocheirus aestivalis* (Fabricius, 1794) and *Heleolaccocoris ovatus*, respectively, most likely represent such cases. Especially since (TGGATAGGTG)n – the motif found in *A. hainanensis* – was recovered at the third position for *A. aestivalis*, suggesting the two *Aphelocheirus* species share the same motif, while the decamer (TGTGCCTCTC)n likely represents an abundant repetitive element.

The phylogenetic relationships within Nepomorpha have been the subject of prolonged debate. While the monophyly of Nepomorpha has been generally supported by morphological and molecular studies (Hebsgaard et al. 2004; Ye et al. 2020; Wang et al. 2021), an early mitochondrial genome study suggested that Pleoidea was not part of Nepomorpha (Hua et al. 2009). This controversial result was subsequently attributed to inadequate taxon sampling, and in particular, long-branch attraction (LBA) between the distant outgroup taxa and Pleoidea, as well as LBA among taxa in the ingroup, which made Nepomorpha appear to be polyphyletic (Li et al. 2014). A close relationship between Notonectoidea and Pleoidea has been consistently recovered in phylogenetic studies, whether Pleoidea is treated as a separate superfamily or as part of an expanded Notonectoidea (Hebsgaard et al. 2004; Li et al. 2014; Ye et al. 2020; Wang et al. 2021). Brożek (2014) similarly supported the monophyly of Pleidae + Helotrephidae and their relationship to Notonectidae based on mouthpart morphology. The sharing of an identical derived decamer across both superfamilies suggests that this motif originated once in their common ancestor. This might represent a novel type of molecular synapomorphy for the Notonectoidea + Pleoidea clade.

**Figure 1.**
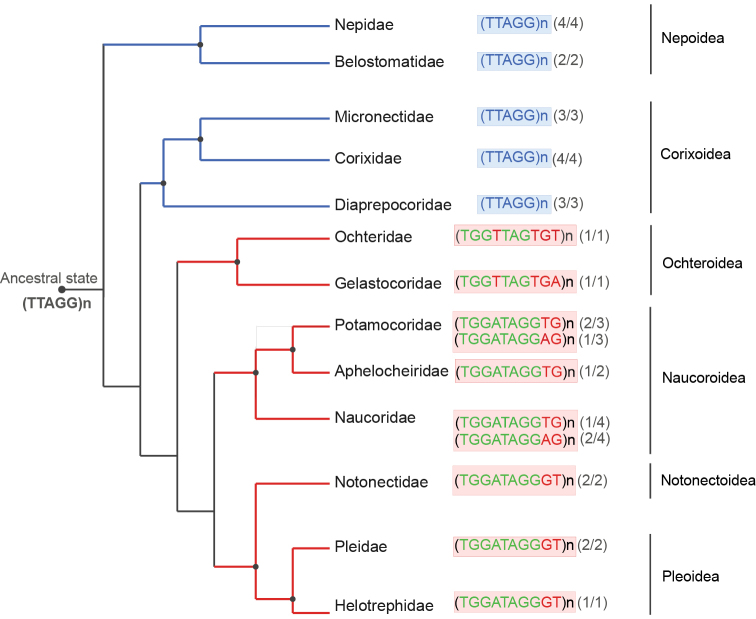
The candidate telomeric repeats identified by genome datasets analysis, mapped on the phylogeny of Nepomorpha (according to Ye et al. 2020 after Wang et al. 2021). For each family, the numbers in brackets indicate the number of datasets in which TREW identified the respective sequence as the best candidate telomeric motif, followed by the total number of analysed datasets for that family. Red lines on the tree mark families with decamer telomeric sequences. The core sequence (TGGATAGG) shared by most of the candidate decamers is in green, and the nucleotide substitutions are in red.

The identity of the basal nepomorphan lineage has been debated, but most phylogenetic hypotheses agree that Nepoidea and Corixoidea represent the two successive early-diverging branches. Morphological studies (Parsons 1966; Popov 1971; Rieger 1976; Mahner 1993) and combined morphological-molecular analyses (Hebsgaard et al. 2004; Ye et al. 2020) typically place Nepoidea as sister to all remaining Nepomorpha, with Corixoidea as the second branch, whereas analyses based primarily on molecular data recover the reverse order, with Corixoidea basal and Nepoidea second (Li et al. 2014; Wang et al. 2016, 2019, 2021; Liu et al. 2018). The only notable exception is Hua et al. (2009), who placed Nepoidea in a derived position sister to Ochteroidea, though this result has been questioned on methodological grounds (Li et al. 2014). Our finding that (TTAGG)n is retained in both Nepoidea and Corixoidea while derived decamers occur in the remaining superfamilies is congruent with most phylogenetic hypotheses and supports (TTAGG)n as the ancestral telomeric motif for Nepomorpha, with 10 bp motifs having evolved in the more derived clades (Fig. 1).

The status of Aphelocheiroidea (comprising Aphelocheiridae and Potamocoridae) has fluctuated between treatment as part of Naucoroidea and as a separate superfamily. Hebsgaard et al. (2004) proposed elevating Aphelocheiroidea based on the strong support for Aphelocheiridae + Potamocoridae as a clade distinct from Naucoridae. In our telomeric data the decamer (TGGATAGGTG)n was retrieved for at least one species of each of the three families: Aphelocheiridae, Potamocoridae, and Naucoridae, supporting the close relationship between these three families as has been suggested previously (Wang et al. 2021; Ye et al. 2020).

In respect to the placement of Ochteroidea (Ochteridae + Gelastocoridae), all recent phylogenetic analyses, support this superfamily as monophyletic and nested within Nepomorpha (Hebsgaard et al. 2004; Ye et al. 2020; Wang et al. 2021). Our data show that both families have related decamers differing only by a single nucleotide, supporting the close relationship of Ochteridae and Gelastocoridae.

Our findings on Nepomorpha are relevant to the broader debate on the origin of 10-bp telomeric motifs in Heteroptera. For the Pentatomomorpha + Cimicomorpha clade (Terheteroptera), Kuznetsova et al. (2025) proposed two alternative hypotheses: under hypothesis 1, the (TTAGGGATGG)n motif arose independently in the common ancestor of Pentatomomorpha and in the common ancestor of all Cimicomorpha excluding Reduvioidea; under hypothesis 2, this decamer was already present in the common ancestor of Terheteroptera, and the canonical (TTAGG)n in Reduviidae represents a secondary reversion. Subsequently, Stoianova et al. (2025) discovered a new 10-bp motif, (TTAGAGGTGG)n, in the gerromorphan *Gerris lacustris*, extending the occurrence of decameric telomeres beyond Terheteroptera to the basal infraorder Gerromorpha. Noting that all four 10-bp motifs then known in Heteroptera (excluding the new motifs reported here) differ only by single nucleotide substitutions at positions 5, 7, 8 and 10, Stoianova et al. (2025) proposed two broader scenarios: either a single 10-bp motif arose early in Heteroptera and subsequently diverged across infraorders, or each infraorder independently acquired the decameric repeat through duplication of the ancestral pentameric TTAGG template in the telomerase RNA followed by the accumulation of substitutions – representing convergent evolution in motif length rather than sequence. Stoianova et al. (2025) noted that distinguishing between these scenarios requires data from basal families within each infraorder: if the earliest-diverging taxa retain (TTAGG)n while 10-bp motifs appear only in more derived lineages, this pattern would strongly support independent evolution. They explicitly noted the need for data from Nepomorpha beyond the basal superfamily Nepoidea. The pattern in Cimicomorpha is consistent with independent evolution: the basal family Reduviidae retains the ancestral (TTAGG)n (Pita et al. 2016; Grozeva et al. 2019; Gapon et al. 2021; Kuznetsova et al. 2025) while the 10-bp motif has so far been reported only in the more derived families Cimicidae, Anthocoridae and Miridae (Stoianova et al. 2024; Kuznetsova et al. 2025). In Pentatomomorpha, however, two species of the basal family Aradidae already possess the 10-bp motif (TTAGGGATGG)n (Stoianova et al. 2025), contrary to the expectation that basal lineages should retain the ancestral pentamer. Our Nepomorpha data directly address the gap identified by Stoianova et al. (2025). The retention of (TTAGG)n in the two basal superfamilies Nepoidea and Corixoidea and the restriction of decamers to the more derived Ochteroidea, Naucoroidea, Notonectoidea and Pleoidea indicate that the 10-bp motif in Nepomorpha arose after the divergence of the basal lineages, rather than being inherited from a heteropteran common ancestor. Furthermore, the Nepomorpha decamers share a core sequence TGGATAGG that is absent from the known decamers of other infraorders – the Terheteroptera motifs contain the core TTAGGG while the Gerromorpha motif (TTAGAGGTGG)n differs at yet other positions – consistent with independent derivation in each infraorder. Taken together, the pattern observed in Cimicomorpha and now in Nepomorpha – ancestral pentamer in basal lineages, derived decamers restricted to more derived clades – favours hypothesis 1 of Kuznetsova et al. (2025) and the convergent-evolution scenario of Stoianova et al. (2025), although the situation in Pentatomomorpha, where the basal family Aradidae already possesses the decameric motif, complicates this interpretation. However, it should be noted that the position of Aradidae as the earliest-diverging extant pentatomomorphan lineage does not preclude acquisition of the 10-bp motif along the Aradidae stem after its divergence from other Pentatomomorpha; extinct stem-group lineages that might have retained the ancestral pentamer are unavailable for study, and the evolutionary history of telomeric motifs along this branch remains unresolved.

Beyond the evolutionary implications, our results provide an empirical assessment of TREW as a tool for telomeric motif discovery from short-read data. The successful recovery of (TTAGG)n from all six Nepoidea datasets – the only superfamily with prior FISH-confirmed telomeres – validates the method’s ability to detect motifs in archival short-read data. More importantly, the phylogenetic coherence of the novel results argues strongly for their biological validity: the uniform recovery of (TTAGG)n across all ten Corixoidea datasets spanning three families and eight species, and of (TGGATAGGGT)n across all five Notonectoidea-Pleoidea datasets spanning three families, would be difficult to explain as systematic artefacts. Similarly, the recovery of very similar decamers differing by single nucleotides from taxonomically related lineages – such as (TGGTTAGTGA)n and (TGGTTAGTGT)n from the two Ochteroidea families, or (TGGATAGGTG)n and (TGGATAGGAG)n from Naucoroidea – is consistent with shared ancestry rather than random noise. The few anomalous results, such as the low-complexity decamer (TGTGCCTCTC)n from *Aphelocheirus aestivalis* and the pentanucleotide (GGATT)n from *Heleolaccocoris ovatus*, likely represent satellite or other repetitive sequences misidentified as telomeric candidates, highlighting a known limitation of k-mer-based approaches when applied to single datasets without replication. Overall, TREW appears well suited for broad taxonomic surveys of telomeric composition using short-read data, particularly when results can be evaluated in a phylogenetic framework where consistency across related taxa serves as an internal measure of reliability. However, FISH or chromosome-level genome assembly validation remains necessary to confirm the chromosomal localization of the candidate motifs identified here.

## Conclusions

Our survey of telomeric sequences across Nepomorpha reveals a phylogenetically structured pattern that is congruent with most current hypotheses of nepomorphan relationships. The ancestral (TTAGG)n is retained in the two basal superfamilies, Nepoidea and Corixoidea, while derived decamers have evolved in the remaining superfamilies. The sharing of an identical derived motif (TGGATAGGGT)n between Notonectoidea and Pleoidea provides additional support for their close phylogenetic relationship and might represent a novel synapomorphy for this clade. Telomeric sequence data thus offer a new class of phylogenetic characters for investigating evolutionary relationships within Nepomorpha and complement traditional morphological and sequence-based approaches.

## Additional information

### Artificial Intelligence (AI) use

The authors accept full responsibility for the content of the manuscript, including the disclosure of any use of AI.

Regarding the use of AI in the preparation of this manuscript, the authors declare the following: GPT

Used for: Language, style and writing

GPT was used for grammar correction and style corrections in the text.

### Author ORCIDs

D. Stoianova https://orcid.org/0000-0001-8040-7158

G. Karagyan https://orcid.org/0000-0001-7708-2145

S. Grozeva https://orcid.org/0000-0003-3196-8068

### Data availability

All of the data that support the findings of this study are available in the main text or Supplementary Information.

## Supplementary materials

Supplementary material 1 Candidate sequences Data type csv Explanation note Candidate telomeric repeat motifs identified by TREW from 32 whole-genome short-read FASTQ datasets representing 24 Nepomorpha genera from all six superfamilies, including NCBI SRA accession numbers, motif sequences, repeat counts, and ranking among top candidates. https://binary.pensoft.net/file/1723367This dataset is made available under the Open Database License (http://opendatacommons.org/licenses/odbl/1.0/). The Open Database License (ODbL) is a license agreement intended to allow users to freely share, modify, and use this Dataset while maintaining this same freedom for others, provided that the original source and author(s) are credited. Desislava Stoianova, Snejana Grozeva

Supplementary material 2 Metadata Data type csv Explanation note Metadata for Suppl. material 1: table SS1, including column names and their definitions. https://binary.pensoft.net/file/1723368This dataset is made available under the Open Database License (http://opendatacommons.org/licenses/odbl/1.0/). The Open Database License (ODbL) is a license agreement intended to allow users to freely share, modify, and use this Dataset while maintaining this same freedom for others, provided that the original source and author(s) are credited. Desislava Stoianova, Snejana Grozeva
